# Mapping the mRS Into the EQ-5D-5L in Patients With Ischemic Stroke

**DOI:** 10.1161/STROKEAHA.126.055741

**Published:** 2026-04-29

**Authors:** Florentina M.E. Pinckaers, Janneke P.C. Grutters, Silvia M.A.A. Evers, Hester F. Lingsma, Hieronymus D. Boogaarts, Diederik W.J. Dippel, Alida A. Postma, Aad van der Lugt, Charles B.L.M. Majoie, Yvo B.W.E.M. Roos, Robert J. van Oostenbrugge, Wim H. van Zwam, Andrea Gabrio

**Affiliations:** 1 Department of Radiology and Nuclear Medicine (F.M.E.P., A.A.P., W.H.v.Z.), Maastricht University Medical Centre, the Netherlands.; 2 Department of Neurology (R.J.v.O.), Maastricht University Medical Centre, the Netherlands.; 3 Cardiovascular Research Institute (CARIM) (F.M.E.P., R.J.v.O., W.H.v.Z.), Maastricht University, the Netherlands.; 4 Care and Public Health Research Institute (CAPHRI) (F.M.E.P., S.M.A.A.E., A.G.), Maastricht University, the Netherlands.; 5 Department of Health Services Research (S.M.A.A.E.), Maastricht University, the Netherlands.; 6 Department of Methodology and Statistics (A.G.), Maastricht University, the Netherlands.; 7 Mental Health and Neuroscience Research Institute (MHENS) (A.A.P.), Maastricht University, the Netherlands.; 8 Science IQ Health, Radboudumc, Nijmegen, the Netherlands (J.P.C.G.).; 9 Centre of Economic Evaluation and Machine Learning, Trimbos Institute, Utrecht, the Netherlands (S.M.A.A.E.).; 10 Department of Public Health (H.F.L.), Erasmus MC University Medical Center, Rotterdam, the Netherlands.; 11 Department of Neurology (D.W.J.D.), Erasmus MC University Medical Center, Rotterdam, the Netherlands.; 12 Department of Radiology (A.v.d.L.), Erasmus MC University Medical Center, Rotterdam, the Netherlands.; 13 Department of Neurosurgery, Radboudumc, Nijmegen, the Netherlands (H.D.B.).; 14 Department of Radiology and Nuclear Medicine (C.B.L.M.M.), Amsterdam UMC, the Netherlands.; 15 Department of Neurology (Y.B.W.E.M.R.), Amsterdam UMC, the Netherlands.

**Keywords:** algorithm, cost-benefit analysis, outcome assessment, health care, quality-adjusted life years, quality of life, stroke, treatment outcome

## Abstract

**BACKGROUND:**

Mapping concerns the cross-walking of one health-related quality of life instrument to another. This study aimed to translate the modified Rankin Scale (mRS) score at 3 months after ischemic stroke into 5-level version of the EQ-5D (EQ-5D-5L)–based utility values, which may be used for the derivation of quality-adjusted life years for cost-effectiveness analyses.

**METHODS:**

Data from 3 Dutch multicenter phase III clinical trials in ischemic stroke were pooled (MR CLEAN [Multicenter Randomized Clinical Trial of Endovascular Treatment for Acute Ischemic Stroke in the Netherlands]-NO-IV, MR CLEAN-MED, and MR CLEAN-LATE). The mRS and EQ-5D-5L were assessed by telephone interview at 3 months poststroke. The correlation (Spearman coefficient) between the mRS and each EQ-5D-5L domain was assessed. Various direct and indirect mapping algorithms were explored. Model performance (predictive ability) was assessed using bootstrapping in the full testing set (mRS score 0–5) and in patients with independent (0–2) and dependent (3–5) mRS scores separately. Results were validated in an external validation set derived from a cross-sectional Dutch study. Final utility estimates per mRS score were assessed in the pooled trial and external validation data set.

**RESULTS:**

The EQ-5D-5L dimensions self-care, daily activities, and mobility demonstrated the strongest correlation to the mRS (*ρ*=0.46–0.70). Correlation coefficients of the pain and anxiety dimensions were relatively poor (*ρ*=0.37–0.45). Indirect mapping with a multinomial logit model was identified as the preferred mapping algorithm. In the pooled data set, the median (interquartile range) age was 71 (62–79), and 56% of patients were male. Mean (SD) fitted utility values per mRS score were; mRS score 0: 0.947 (0.004); mRS score 1: 0.860 (0.009); mRS score 2: 0.758 (0.013); mRS score 3: 0.597 (0.017); mRS score 4: 0.352 (0.017); and mRS score 5: 0.147 (0.017).

**CONCLUSIONS:**

The mean utility weights provided in this study may be used for the direct estimation of quality-adjusted life years from mRS scores. Furthermore, the multinomial logit model may be used to derive utility values from new data sets with different country-specific utility tariffs. Our results are ideally suited for application in cost-effectiveness analyses.

The modified Rankin Scale (mRS) is the most widely used primary outcome measure in acute stroke intervention trials. However, decisions on reimbursement are typically based on economic evaluations that rely on the use of quality-adjusted life years as a summary measure of health outcomes.^1,2^ Unfortunately, many studies do not include utility measures such as the EQ-5D^3^ within their research protocols, which are, in principle, a mandatory source for the computation of individual quality-adjusted life year values.^4^ To address this problem, several studies have created a value set for use in further analyses by mapping the mRS scores into utility values.^4–8^ These value sets have also been used in the creation of a utility-weighted mRS, which has been proposed and applied as an alternative primary outcome measure to the mRS.^5,9^

Previous studies concerning the mapping of the mRS scores into the EQ-5D have all used the 3-level version of the EQ-5D (EQ-5D-3L).^4–8^ However, the EQ-5D-3L is known to suffer from ceiling effects and overestimation of self-reported health issues.^10^ The newer 5-level version of the EQ-5D (EQ-5D-5L) has been shown to be superior to the EQ-5D-3L with respect to various measurement properties, including sensitivity and precision in health status measurement.^11^ As a result, several countries now recommend the usage of the EQ-5D-5L over the EQ-5D-3L.^12^

The mapping of mRS scores into an EQ-5D-5L value set has not yet been reported. This study aims to develop an algorithm for the estimation of EQ-5D-5L–derived utility values from mRS scores at 3 months poststroke using pooled data from 3 Dutch ischemic stroke intervention trials.

## Methods

This article was prepared in accordance with the Mapping onto Preference-Based Measures Reporting Standards,^13^ the ISPOR (The Professional Society for Health Economics and Outcomes Research) Task Force report on mapping,^14^ and the NICE (National Institute for Health and Care Excellence) best practice recommendations on mapping.^15^

### Data Availability Statement

Deidentified data can be made available on reasonable request. Information about analytic methods and R scripts/output files will be shared on reasonable request.

### Study Data Set

Data from 3 multicenter phase III clinical trials were pooled: MR CLEAN (Multicenter Randomized Clinical Trial of Endovascular Treatment for Acute Ischemic Stroke in the Netherlands)-NO-IV, MR CLEAN-MED, and MR CLEAN-LATE.^16–18^ All 3 trials considered patients with a large vessel occlusion of the anterior circulation. General inclusion criteria were age ≥18 years; a National Institutes of Health Stroke Scale score of ≥2; and an occlusion of the intracranial internal carotid artery, carotid artery terminus, or middle cerebral artery (M1 or proximal M2 segment). All 3 trials excluded patients with prestroke dependence (defined as mRS score >2). The trials included patients between January 2018 and January 2022. A central medical ethics committee at Erasmus MC, University Medical Center Rotterdam (Rotterdam, the Netherlands) approved the trials. Written (deferred) informed consent was obtained from patients or their legal representatives.

For the present study, patients who were deceased at 3 months poststroke or in whom the EQ-5D-5L score was missing were excluded (Figure).

**Figure.**
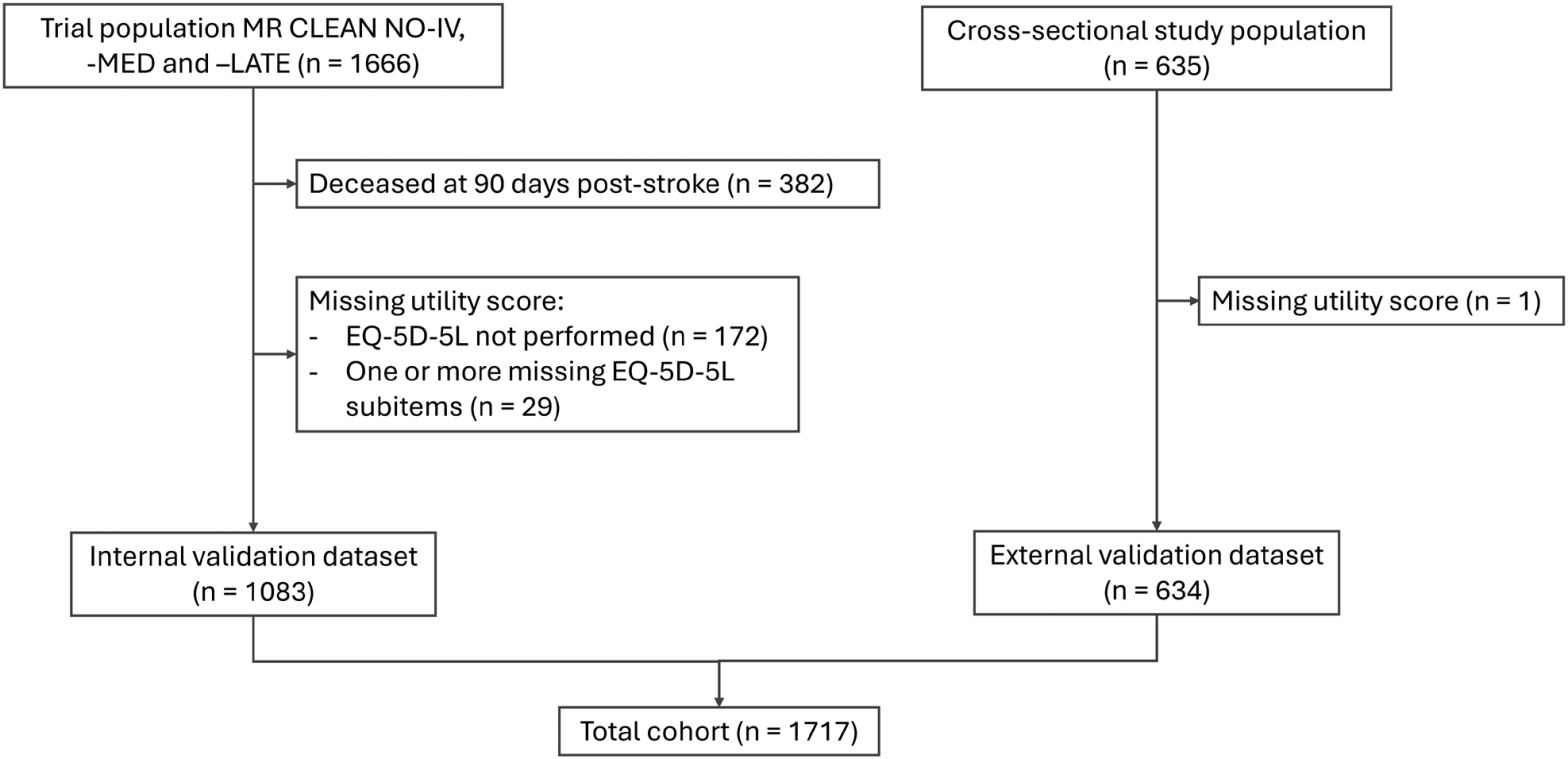
**Inclusion flowchart.** EQ-5D-5L indicates 5-level version of the EQ-5D; and MR CLEAN, Multicenter Randomized Clinical Trial of Endovascular Treatment for Acute Ischemic Stroke in the Netherlands

### Instruments

At 90 days poststroke, the mRS score and EQ-5D-5L were assessed by a trained research nurse during a single telephone interview with the patient. If the patient was unable to partake in the interview, their representative answered the questions on their behalf instead.

The mRS score measures the degree of dependence in daily life and provides a functional outcome score ranging from 0 (no symptoms) to 6 (deceased).^19^

The EQ-5D-5L is a preference-based measure in which 5 dimensions (mobility, self-care, usual activities, pain/discomfort, and anxiety/depression) are graded on 5 severity levels (no, slight, moderate, severe, extreme problems).^3^ It is a generic health-related quality of life instrument capturing both motor and nonmotor outcomes, and is currently one of the most widely used patient-reported outcome measures internationally.^20,21^ EQ-5D-5L health states are defined by a 5-number sequence describing the score per health domain, with 11 111 representing perfect health and 55 555 representing the worst possible health state. A Dutch, composite time trade-off–based value set was used to derive utilities from EQ-5D-5L responses.^22^ The theoretical range of the utility values was −0.446 to 1 (full health). The utility of deceased patients corresponds to 0, and negative utilities therefore represent health states worse than death.

### Statistical Analysis

Descriptive statistics for the mRS score and EQ-5D-5L were obtained. To check whether a sufficiently high level of overlap between each individual EQ-5D dimension score and the mRS score existed, the association between the 2 measures in the data set was assessed by means of Spearman correlation coefficients. Sufficient level of overlap was judged based on a correlation value of at least 0.3, taking into account the potential skewness and non-normality of the data.^23^

All statistical analyses were performed in R version 4.5.0.^24^

### Model Specification

The empirical distribution of utilities is characterized by several non-normal features.^14^ First, the maximum theoretical utility value is 1 (full health), at which point the distribution is typically characterized by a large spike. In addition, the distribution shows different degrees of skewness, multimodality, and gaps in the observed range of values, which further complicates the application of any simple regression method for mapping the mRS score into the EQ-5D.^14^ We explored several direct and indirect mapping approaches to compare the performance of different methods and to identify the best-performing model among those assessed.

In direct mapping approaches, EQ-5D-5L–derived utilities are regressed on a series of potentially associated independent variables with a substantial predictive power towards the outcome. Based on previous literature, 5 statistical direct mapping models were selected and tested on the data: ordinary least squares (OLS) regression; censored Tobit regression; zero-inflated gamma regression (with a logarithmic function as link function); hurdle model gamma regression (with a logarithmic function as link function); and an adjusted limited dependent variable mixture model.^14^

OLS regression is the simplest and most often used technique in mapping.^25^ It assumes a linear relationship between the dependent and independent variables and normally distributed error terms. Because these assumptions are in clear conflict with the previously described distributional features of utility values, the application of OLS regression for mapping is likely to result in systematic bias, underestimation of utility values for mild health states, and overestimation for more severe health states.^26^ Furthermore, OLS regression may result in the prediction of values outside the upper and lower boundaries of the EQ-5D-5L value set. A second approach is given by a censored Tobit model, which allows to take into account the existence of the upper boundary of 1 and the substantial spike of observations occurring at the full health point.^27,28^ A third and fourth approach consists of the so-called 2-part models, either by fitting a zero-inflated or hurdle model gamma regression on disutility values (1-utility), which represent an alternative way to address the same non-normal features in the data.^29^ However, similar issues to OLS regression with predictions at the upper and lower ends of the distribution have been reported for both Tobit and 2-part models.^14^ Finally, the adjusted limited dependent variable mixture model approach allows for the modeling of complex nonstandard distributions, accounting for latent classes, multimodality, minimum and maximum utility values, and gaps.^28^ These characteristics are particularly desirable for mapping, as they are likely to improve model fit and predictive accuracy compared with standard regression approaches.^28^ The adjusted limited dependent variable mixture model was fitted using the *aldvmm* R package, using various optimization methods (Broyden-Fletcher-Goldfarb-Shanno, conjugate gradient, Nelder-Mead, nlminb, Rcgmin, and Rvmmin), multiple initialization options (zero, constant, and sann), and different numbers of mixture models (ie, components).^30^ For illustration, histograms of the models’ distributional assumptions are shown in Figure S1.

In an indirect mapping approach (ie, response mapping), separate regression models are fitted to each dimension of the EQ-5D-5L to estimate the probability of being in each of the 5 response levels. The predicted utility value is then calculated by multiplying the predicted response level probabilities for each dimension with the corresponding values of the value set (see Appendix II: Indirect Mapping Algorithm in the Supplemental Material for a more in-depth description of the approach). The main advantage of an indirect mapping approach is that different value sets can be applied to the predicted response level probabilities, allowing for the derivation of country-specific utility values. We applied multinomial logit and ordered probit regression as indirect mapping methods.^4,27,31^ A possible issue in fitting indirect mapping models is that certain theoretical combinations of mRS scores and EQ-5D-5L responses may, in fact, not occur in the observed sample (eg, an mRS score of 0 and severe problems on the mobility dimension), in which case the corresponding coefficient estimates from the fitted model cannot be obtained. This drawback can be overcome by using neural networks when fitting the multinomial logit model (eg, using the *nnet* R package)^32^ or by using Bayesian functions when fitting the ordered probit model (eg, using the *arm* R package).^33^

As age and sex are often relevant sociodemographic characteristics that affect utility values,^34^ they were included as covariates in all assessed models. These covariates had no missing data.

### Internal Validation of Model Performance

Optimism corrected bootstrapping using Harrell’s bias correction was used to assess model performance.^35^ A model was first fitted to the entire data set to estimate its predictive ability *Performance*_*total*_. Next, 100 bootstrap samples were taken from the original data. The model was fitted in each bootstrap sample, and the in-sample performance, *Performance*_*boot*_ was assessed. Each bootstrap model was also applied to the original data set to derive *Performance*_*original*_. The optimism constitutes the mean of *Performance*_*boot*_ minus *Performance*_*original*_. The optimism corrected bootstrap is the *Performance*_*total*_ minus the optimism. We used the mean absolute error and the root mean squared error as measures of predictive ability. Summary statistics (bootstrapped mean and 95% CI) and plots of the observed versus predicted values were also evaluated.

As previously stated, mapping algorithms may be able to reliably predict mean scores but perform poorly at the low and high ends of the spectrum. For this reason, model performance was also assessed separately for independent (mRS scores 0–2) and dependent states (mRS scores 3–5).

### External Validation

To assess the generalizability of our results to a different patient population, we used a data set from a previously published study on cost- and utility estimates after ischemic stroke.^34^ This previous study combined MR CLEAN-LATE data with a cross-sectional single-center patient cohort. For the present external validation set, MR CLEAN-LATE records were excluded to avoid patient overlap with the internal validation sample. Records with missing EQ-5D-5L data were likewise excluded (Figure).

As in the internal validation data set, patients or their representatives participated in a telephone interview with a trained research nurse in which the mRS score and EQ-5D-5L were scored. The interview could take place at any moment up to 2 years poststroke.

Model performance was assessed in the full external validation data set, as well as in independent (mRS scores 0–2) and dependent (mRS scores 3–5) subgroups.

### Estimation of Utility Weights in All Available Data

Based on the results in the internal and external validation data sets, the preferred modeling method was selected. As a final step, all models were refit on all available data (combined internal and external validation data set). The model coefficients and the corresponding fitted mean utility weights per mRS score are reported.

## Results

The 3 trials included a total of 1666 patients, of which 382 were deceased at 3 months poststroke. Of the remaining 1284 records, 201 patients had a missing utility score, resulting in an included cohort of 1083 patients. The external validation set contained 635 unique records, of which 1 patient was excluded due to missing EQ-5D-5L responses (Figure).

Baseline characteristics of the internal and external validation sets are described in Table 1. In the internal and external validation sets, the median (interquartile range) age was 71 (61–78) and 72 (63–79) years, respectively, and 54% and 56% of patients were male.

**Table 1.**
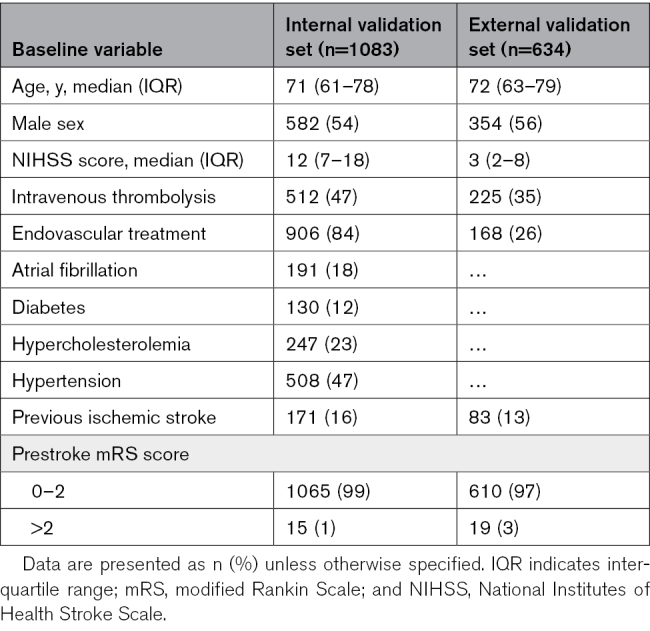
Patient Characteristics

In Tables 2 and 3, the distribution of mRS and EQ-5D-5L scores is described. In the internal and external validation data set, respectively, 65% and 75% of patients were functionally independent at 3 months poststroke (Table 2). The correlation between the mRS and EQ-5D-5L dimensions is described in Table 4. The correlation (Spearman coefficient) was highest for self-care, daily activities, and mobility, whereas the correlation with anxiety was relatively poor.

**Table 2.**
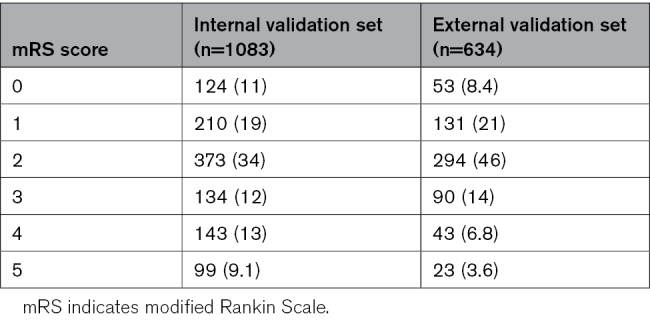
mRS Scores in the Internal and External Validation Sets, n (%)

**Table 3.**
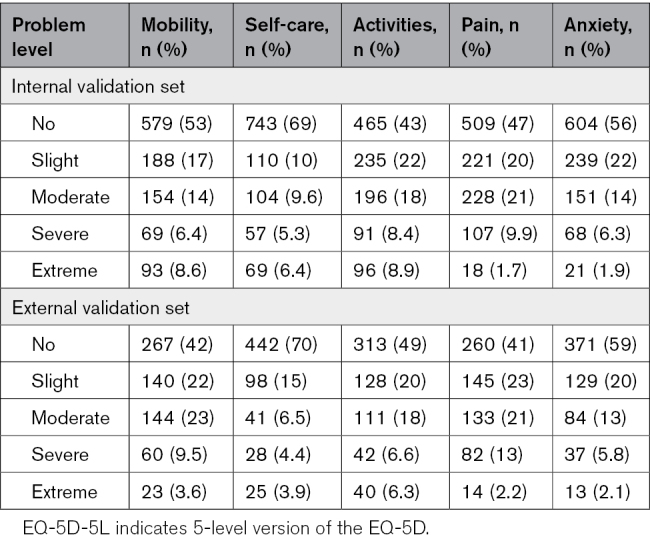
EQ-5D-5L Response Levels in the Internal and External Validation Sets

**Table 4.**
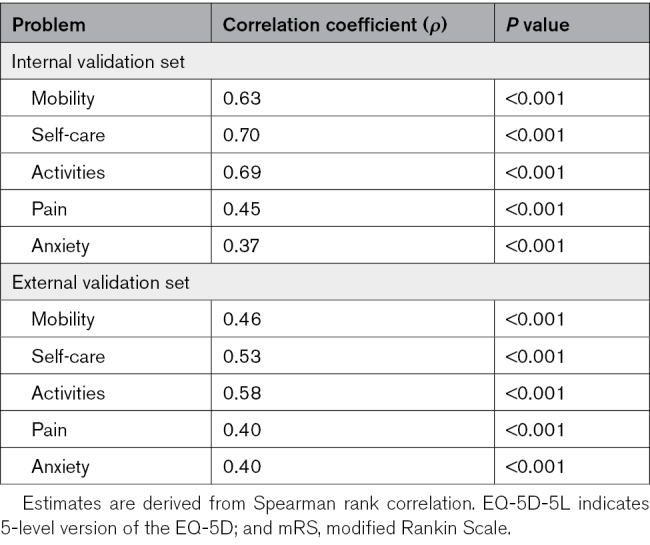
Correlation Between the mRS Score and EQ-5D-5L Dimensions in the Internal and External Validation Dataset

The distribution of EQ-5D-5L–derived utility values was left-skewed (Figure S2). In both the internal and external validation sets, the maximum utility value was 1, and the minimum utility value was −0.4463.

In the internal and external validation sets, respectively, 43% and 14% of mRS scores and EQ-5D-5L responses were provided by the proxy.

### Model Performance

Model performance in the complete internal validation data set and in independent and dependent subgroups is shown in Table 5. Table 6 shows model performance in the complete external validation set and in functional outcome subgroups.

**Table 5.**
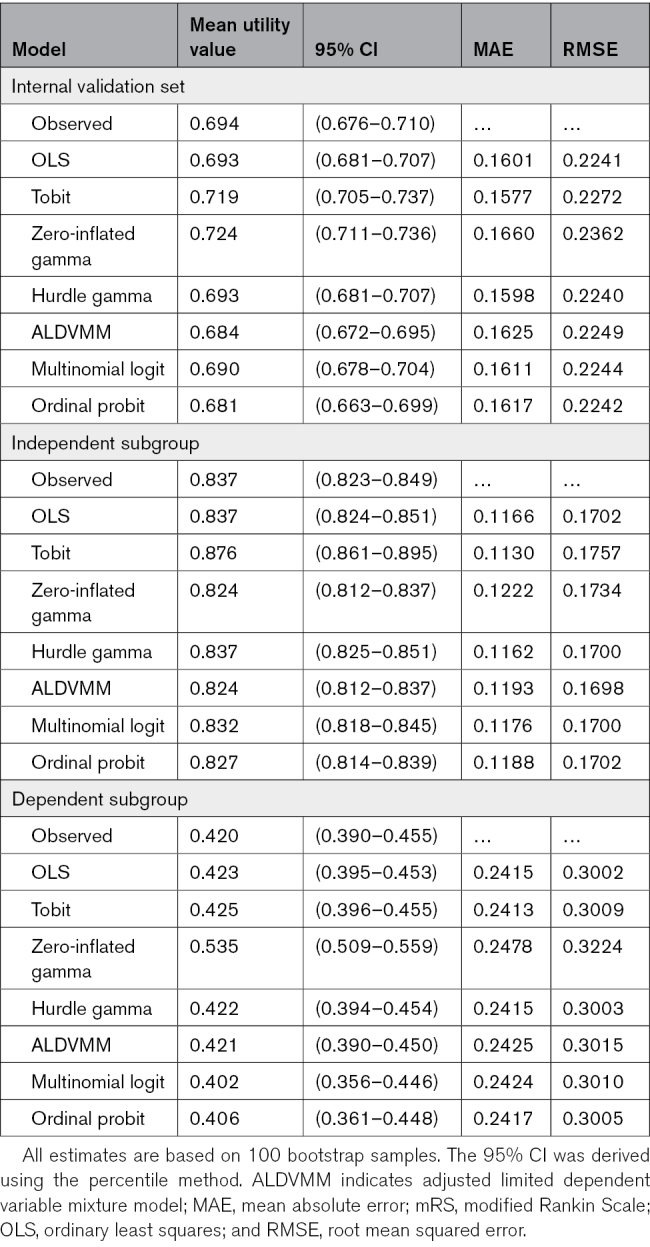
Bootstrapped Descriptives and Optimism Corrected Bootstrap Estimates of Model Performance in the Entire Internal Validation Set and in Subgroups of mRS Scores

**Table 6.**
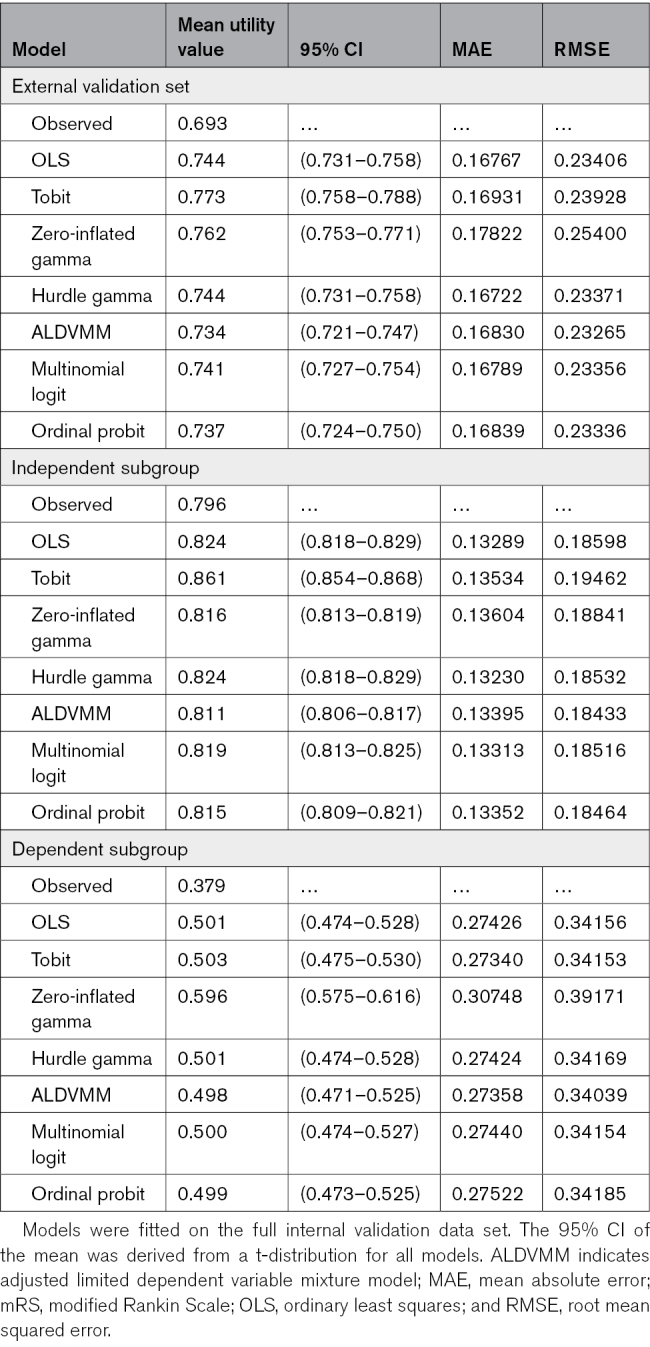
Bootstrapped Descriptives and Optimism Corrected Bootstrap Estimates of Model Performance in the Entire External Validation Set and in Subgroups of mRS Scores

The models with the most consistent performance on mean absolute error and root mean squared error in both the internal and external validation sets were the hurdle gamma and multinomial logit model (Tables 5 and 6). If we consider the root mean squared error (which penalizes larger outliers), the hurdle gamma model demonstrated a slightly better performance in the internal validation set, whereas the multinomial logit model was preferred in the external validation set. The mean absolute error favored the hurdle gamma model in both the internal and external validation sets. Model performance was generally worse in the dependent subgroup. Because the multinomial logit model may be applied to new data sets using country-specific utility tariffs, this model was identified as the preferred model among those assessed. A histogram showing the difference between the observed values and the values predicted by the multinomial logit model is presented in Figure S3.

### Estimation of Utility Weights

Table 7 describes the observed and fitted mean (SD) utility weights per mRS score for all evaluated models in the combined internal and external validation data set. The mean (SD) fitted utility weights per mRS score for the multinomial logit model were; mRS score 0: 0.947 (0.004); mRS score 1: 0.860 (0.009); mRS score 2: 0.758 (0.013); mRS score 3: 0.597 (0.017); mRS score 4: 0.352 (0.017); and mRS score 5: 0.147 (0.017). The model coefficients of all evaluated models are reported in Tables S1 through S7. The multinomial logit model is shared in R files in the Supplemental Material (please see Appendix V: Instructions Pertaining R Files in the Supplemental Material for further information).

**Table 7.**
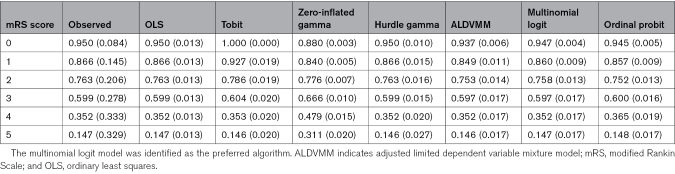
Summary of Observed and Predicted Mean (SD) Utility Values per mRS Score in the Total Data Set

## Discussion

In this study, we have mapped the mRS into the EQ-5D-5L using a range of popular direct and indirect mapping algorithms. Based on performance metrics (mean absolute error and root mean squared error), indirect mapping with a multinomial logit model had the best performance among the evaluated approaches. A key advantage of the model is that it can be applied to new data sets to derive quality-adjusted life years from mRS scores using country-specific utility tariffs, making it ideally suited for application in future cost-effectiveness analyses in stroke when information on health-related quality of life of the target population is not readily available.

Although mRS scores have been mapped to the EQ-5D before, this is the first study to use the 5-level version of the EQ-5D as opposed to the 3-level version.^4–8^ As the EQ-5D-5L has been shown to be superior to the EQ-5D-3L in various respects, including its precision in health status measurement,^11^ this may improve the internal validity of our findings compared with previous studies. Furthermore, most of the studies only reported the arithmetic mean utility per mRS score^5,6,8^ or mean estimates derived from OLS regression (with adjustments for other variables, for example, age).^7^ Although OLS regression is one of the most commonly used mapping methods, it may not be optimal given the peculiar distributional features which are typical of EQ-5D–derived utilities. In our study, the multinomial logit model yielded a better performance and more accurate predictions compared with the OLS model. Considering the accuracy of the predictions of the evaluated models, it is worth noting that the mRS score is an instrument aimed at capturing differences in functional outcomes. It does not encompass the EQ-5D dimensions of pain and anxiety, which is reflected in the poorer correlations between the mRS score and these dimensions. This is in line with previous findings.^4^ Likewise, the EQ-5D-5L is a generic health-related quality of life outcome questionnaire, and it does not capture all patient-reported outcomes relevant to poststroke recovery (eg, cognition, fatigue, and communication).^36^

Only Rivero-Arias et al^4^ have previously evaluated the performance of an indirect mapping algorithm, namely a multinomial logit model. Although the multinomial logit model showed a similar performance compared with OLS regression in their study, the authors noted that the penalty associated with an incorrect prediction is likely higher with the EQ-5D-3L than with the EQ-5D-5L.^4^ In our study using the EQ-5D-5L, the multinomial logit model was the preferred model among our evaluated approaches. The main advantage of indirect mapping models is that the predicted response values can be adapted to new data sets with various country-specific tariffs. For example, the frequently used 3L UK tariff is known to yield notably lower utility values compared with the tariffs of other countries.^37^ As a result, the model coefficients reported in the present study may be used to predict utility values using different value sets, taking possible cultural differences in the valuation of quality of life into account.

Regardless of the applied mapping algorithm, our fitted utility values differed substantially per mRS score. These results provide a further argument against the pooling of mRS score categories in cost-effectiveness analyses. The fitted utility value of the mRS score of 5 (0.147 with the multinomial logit model) was also significantly higher than 0, which is the set utility value that has been applied to this health state in some previous studies.^5^ When directly comparing our fitted values to a previous Dutch study describing mean utilities derived from EQ-5D-3L scores, our estimates are comparable.^6^ Our fitted utility values in the internal and the external validation data set were also rather similar. Model performance metrics were poorer in the dependent subgroup for all evaluated models, which likely reflects a larger variation in utility scores in these patients. Furthermore, in the external validation set, we observed a slight bias in the predictions towards better utility values. This may have been caused by the fact that the external validation set was derived from a single-center patient cohort collected at the Maastricht University Medical Center+, which is situated in an area with a low socioeconomic and health status compared with the rest of the Netherlands.^38^

Key strengths of this study include the combined usage of cross-sectional data with data from 3 Dutch randomized controlled trials with broad and pragmatic inclusion criteria. The data set also included a sufficiently large number of patients who reported poor mRS scores and extreme problems on the EQ-5D-5L (Tables 2 and 3). We evaluated various direct and indirect mapping algorithms with optimism-corrected bootstrapping in an internal validation set, and validated model performance in an external validation set. Finally, we chose to refit the preferred model on all available data to derive model coefficients and utility weights for application in future (cost-effectiveness) research.

A limitation of this study is that 15% of trial patients alive at 3 months poststroke had to be excluded because of missing or incomplete EQ-5D-5L responses. Of these patients, 70% had an mRS score of 3 to 5 (functional dependence). This may have introduced bias in our analyses. Nevertheless, a missingness rate of 15% is in line with those reported in previous studies; for example, Rivero-Arias et al^4^ reported an EQ-5D missingness rate of 24% at 1 month poststroke, and 31% at 6 months poststroke.

Although the sole usage of Dutch data improved the internal validity of our analyses, it may limit generalization to other countries. However, provided that patients in a different setting give similar answers to the EQ-5D-5L questionnaire, the coefficients of our multinomial logit model may be used with a different country-specific tariff. Nevertheless, further validation of our results in different settings and with other country-specific tariffs is recommended. Furthermore, as our study included only ischemic stroke patients, the generalizability to other stroke types needs to be assessed.

## Conclusions

The utility weights provided in this study may be used for the estimation of quality-adjusted life years for cost-effectiveness analyses. Furthermore, our model coefficients may be used to predict utility values in new data sets using country-specific utility tariffs. Further validation of our results in different data sets with other country-specific tariffs is recommended.

## Article Information

### Acknowledgments

The authors thank the MR CLEAN (Multicenter Randomized Clinical Trial of Endovascular Treatment for Acute Ischemic Stroke in the Netherlands)-NO-IV, MR CLEAN-MED, and MR CLEAN-LATE trial investigators for providing the data used in this study.

### Disclosures

The authors report no disclosures related to the content of this manuscript. Dr Grutters reports grants from ZonMw and Zorginstituut Nederland, all paid to the institution. Dr Evers reports compensation from Maastricht University for other services. Dr Postma reports compensation from Maastricht University for other services. Dr van der Lugt reports grants from the Dutch Brain Foundation, GE Healthcare, Health Holland Top Sector Life Sciences & Health, Philips, Medtronic USA, Siemens Healthcare, Trombolytic Science LLC, Dutch Heart Foundation, the Netherlands Organisation for Health Research and Development, Boehringer Ingelheim, Penumbra, and Stryker Corporation, all paid to the institution. Dr Majoie reports grants from the Dutch Heart Foundation, European Commission, Boehringer Ingelheim, and Stryker Corporation, all paid to the institution, and stock holdings in Nico-Lab. Dr Roos reports stock holdings in Nico-Lab. Dr van Zwam reports grants from Penumbra, MicroVention, Johnson and Johnson International, Bayer HealthCare Pharmaceuticals, and Stryker Corporation, all paid to the institution, and compensation from Philips for data and safety monitoring services, all paid to the institution. The other authors report no conflicts.

### Supplemental Material

Supplemental Methods

Tables S1–S7

Figures S1–S3

Supplemental R Files

MAPS Statement

## Supplementary Material
