# Supplementary material for: Mapping the mRS Into the EQ-5D-5L in Patients With Ischemic Stroke
Source: Stroke. 2026 Apr 29;57(6):1658–66. doi: 10.1161/STROKEAHA.126.055741 (PMC13196856; doi:10.1161/STROKEAHA.126.055741)
Supplement: Supplementary file 1 [file str-57-1658-s001.pdf]

SUPPLEMENTAL MATERIAL

Mapping the Modified Rankin Scale (mRS) into the 5-level EQ-5D (EQ-5D-5L) in Ischemic Stroke Patients

Table of Contents

Appendix I: Examples of model distributions 2
Appendix II: Indirect mapping algorithm 3
Appendix III: Additional figures 4
Appendix IV: Coefficients of all evaluated models 5
Appendix V: Instructions pertaining R files 14
Variable description 14
R code sample 15

Appendix I: Examples of model distributions

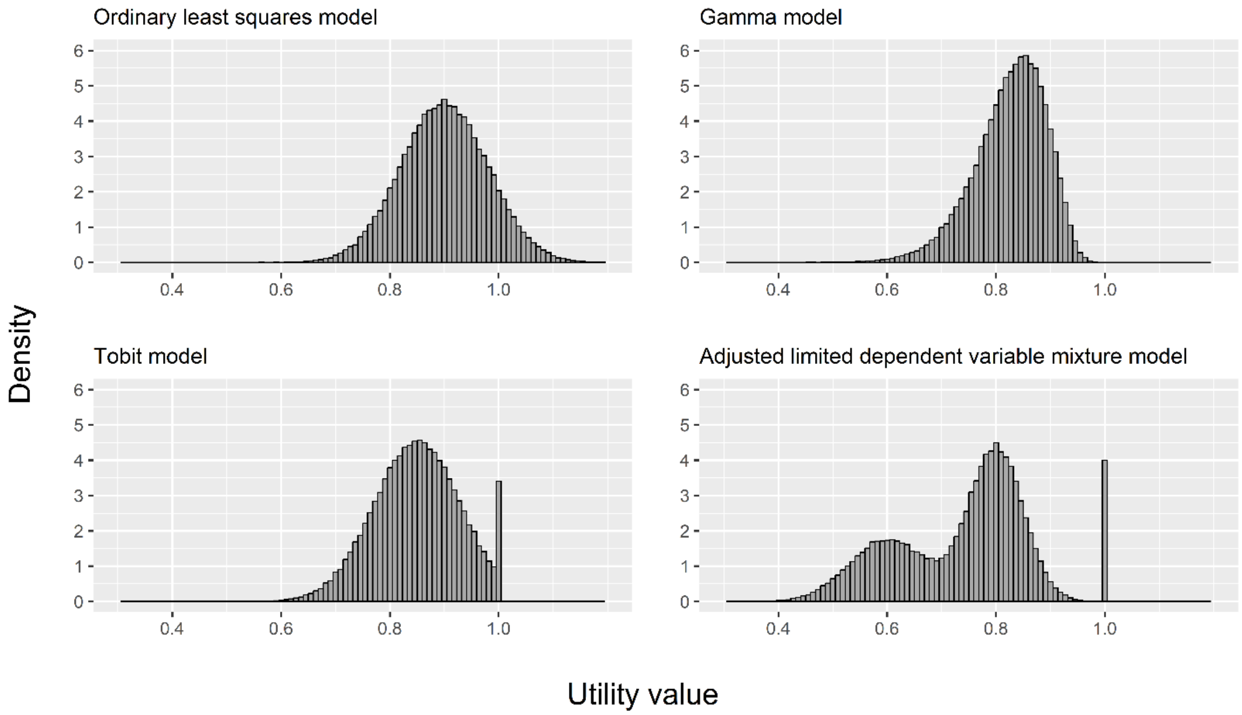

Figure S1: Example histograms of model distributions.

Appendix II: Indirect mapping algorithm

In the indirect mapping algorithm, the predicted utility is calculated by multiplying the predicted response level probabilities for each dimension with the corresponding values of the value set, as demonstrated in the equation below. The following abbreviations are used: mobility (M); self-care (SC); usual activities (UA); pain/discomfort (PD); anxiety/depression (AD). The numbers in the abbreviation indicate the severity level, e.g. M2 is the probability of scoring 2 on the mobility dimension.

$$
\begin{aligned}
\textit{Predicted utility} = {} & 1 - 0.0354544 * M2 - 0.0565962 * M3 - 0.166003 * M4 - 0.2032975 * M5 - \\
& 0.0381079 * SC2 - 0.0605347 * SC3 - 0.1677852 * SC4 - 0.1677852 * SC5 - \\
& 0.0391539 * UA2 - 0.0867559 * UA3 - 0.1924631 * UA4 - 0.1924631 * UA5 - \\
& 0.0658959 * PD2 - 0.0919619 * PD3 - 0.35993 * PD4 - 0.4152142 * PD5 - \\
& 0.069622 * AD2 - 0.1445222 * AD3 - 0.3563913 * AD4 - 0.4206361 * AD5 - \\
& (1 - M1 * SC1 * UA1 * PD1 * AD1) * 0.0469233
\end{aligned}
$$

Appendix III: Additional figures

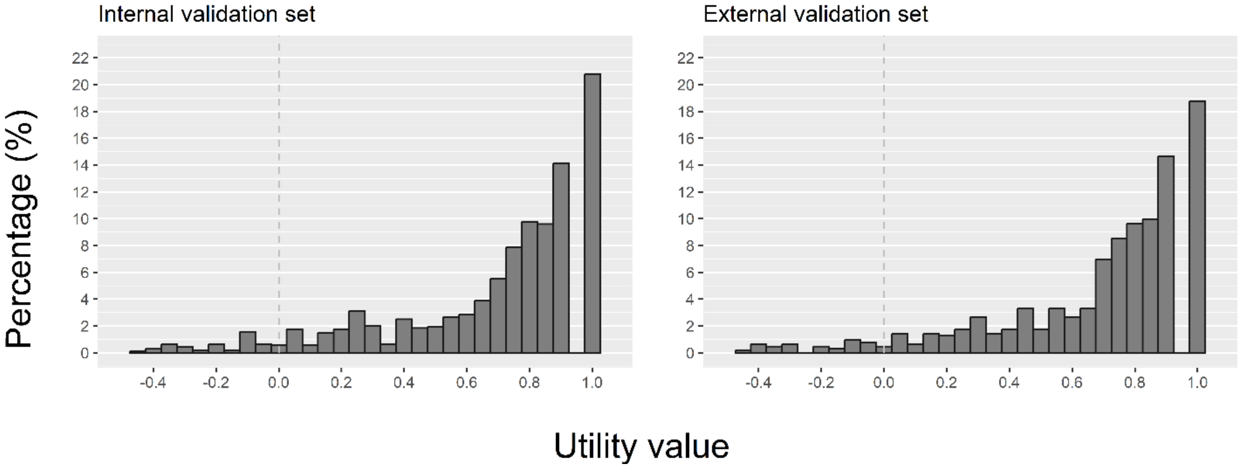

Figure S2: Distribution of utility values in the internal and external validation set.

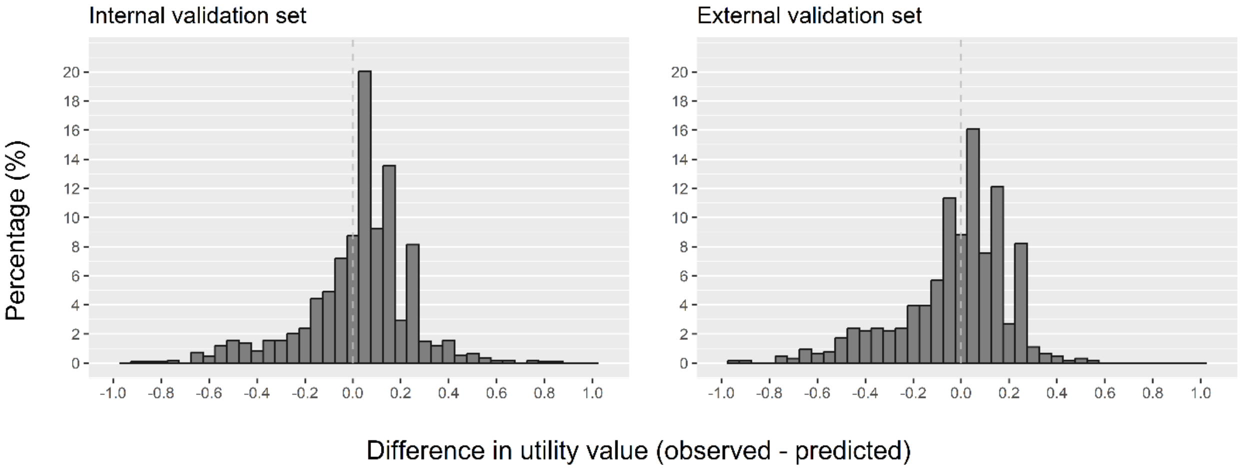

Figure S3: Histogram showing the difference between the observed values and the values predicted by the multinomial logit model in the internal and external validation dataset.

Appendix IV: Coefficients of all evaluated models

Table S1: Parameter estimates of the ordinary least squares model

Parameter	Estimate (SE)	P-value
mRS 1	-0.083 (0.021)	**<.001**
mRS 2	-0.186 (0.019)	**<.001**
mRS 3	-0.348 (0.023)	**<.001**
mRS 4	-0.594 (0.024)	**<.001**
mRS 5	-0.800 (0.027)	**<.001**
Sex	0.026 (0.011)	**.018**
Age	0.000 (0.000)	.766
Intercept	0.924 (0.037)	**<.001**

Abbreviations: mRS=modified Rankin Scale; SE=standard error
Note: mRS 0 is the reference category. Female sex is the reference category. Estimates with P<.05 are printed bold.

Table S2: Parameter estimates of the Tobit model

Parameter	Estimate (SE)	P-value
mRS 1	-0.197 (0.029)	**<.001**
mRS 2	-0.338 (0.027)	**<.001**
mRS 3	-0.518 (0.030)	**<.001**
mRS 4	-0.768 (0.031)	**<.001**
mRS 5	-0.974 (0.034)	**<.001**
Sex	0.039 (0.013)	**.003**
Age	0.000 (0.001)	.882
Intercept 1	1.096 (0.046)	**<.001**
Intercept 2	-1.331 (0.021)	**<.001**

Abbreviations: mRS=modified Rankin Scale; SE=standard error.
Note: mRS 0 is the reference category. Female sex is the reference category. Estimates with P<.05 are printed bold.

Table S3: Parameter estimates of the zero-inflated gamma model

**Parameter**	**Estimate (SE)**	**P-value**
Conditional		
mRS 1	0.287 (0.084)	**<.001**
mRS 2	0.623 (0.079)	**<.001**
mRS 3	1.021 (0.084)	**<.001**
mRS 4	1.464 (0.086)	**<.001**
mRS 5	1.743 (0.092)	**<.001**
Sex	-0.058 (0.031)	.061
Age	0.000 (0.001)	.753
Intercept	-1.837 (0.117)	**<.001**
Zero-inflation		
Intercept	-1.384 (0.060)	**<.001**

Abbreviations: mRS=modified Rankin Scale; SE=standard error.
Note: mRS 0 is the reference category. Female sex is the reference category. Estimates with P<.05 are printed bold.

Table S4: Parameter estimates of the hurdle gamma model

Parameter	Estimate (SE)	P-value
Conditional		
mRS 1	0.287 (0.084)	**<.001**
mRS 2	0.623 (0.079)	**<.001**
mRS 3	1.021 (0.084)	**<.001**
mRS 4	1.464 (0.086)	**<.001**
mRS 5	1.743 (0.092)	**<.001**
Sex	-0.058 (0.031)	.061
Age	0.000 (0.001)	.753
Intercept	-1.837 (0.117)	**<.001**
Zero-inflation		
mRS 1	-1.408 (0.198)	**<.001**
mRS 2	-2.414 (0.195)	**<.001**
mRS 3	-3.842 (0.377)	**<.001**
mRS 4	-5.876 (1.016)	**<.001**
mRS 5	-5.431 (1.018)	**<.001**
Sex	0.494 (0.143)	**<.001**
Age	-0.003 (0.006)	.658
Intercept	0.580 (0.457)	.204

Abbreviations: mRS=modified Rankin Scale; SE=standard error.
Note: mRS 0 is the reference category. Female sex is the reference category. Estimates with P<.05 are printed bold.

Table S5: Parameter estimates of the adjusted limited dependent variable mixture model

Parameter	Estimate (SE)	P-value
**Conditional mean (E[y|X, c])***		
Component 1		
mRS 1	-0.179 (0.065)	**.006**
mRS 2	-0.300 (0.047)	**<.001**
mRS 3	-0.451 (0.061)	**<.001**
mRS 4	-0.706 (0.059)	**<.001**
mRS 5	-0.906 (0.060)	**<.001**
Sex	0.011 (0.016)	.483
Age	0.000 (0.003)	.936
Intercept	1.068 (0.162)	**<.001**
Insigma	-1.395 (0.034)	**<.001**
Component 2		
mRS 1	-0.179 (0.065)	**.006**
mRS 2	-0.300 (0.047)	**<.001**
mRS 3	-0.451 (0.061)	**<.001**
mRS 4	-0.706 (0.059)	**<.001**
mRS 5	-0.906 (0.060)	**<.001**
Sex	0.011 (0.015)	.469
Age	0.000 (0.003)	.937
Intercept	1.068 (0.163)	**<.001**
Insigma	-1.395 (0.034)	**<.001**
**Component probability (P[c|X])****		
Component 1	0.000 (7655.059)	1.000

Abbreviations: mRS=modified Rankin Scale; SE=standard error.
Note: mRS 0 is the reference category. Female sex is the reference category. Estimates with P<.05 are printed bold.
*The conditional mean E[y|X, c] is the expected value of the outcome Y given X and c.
**The component probability P[c|X] is the (average) probability that an observation belongs to component c, given X.

Table S6: Parameter estimates (SE) of the multinomial logit model

	Mobility		*Selfcare*		*Daily activities*		*Pain*		*Anxiety*	
**Parameter**	**Estimate (SE)**	**P-value**	**Estimate (SE)**	**P-value**	**Estimate (SE)**	**P-value**	**Estimate (SE)**	**P-value**	**Estimate (SE)**	**P-value**
Slight problems										
mRS 1	1.701 (0.391)	**<.001**	1.694 (0.748)	**.024**	2.000 (0.438)	**<.001**	0.898 (0.270)	**<.001**	0.729 (0.270)	**.007**
mRS 2	2.348 (0.378)	**<.001**	2.607 (0.722)	**<.001**	2.907 (0.426)	**<.001**	1.485 (0.253)	**<.001**	1.004 (0.253)	**<.001**
mRS 3	2.974 (0.405)	**<.001**	3.897 (0.729)	**<.001**	3.755 (0.460)	**<.001**	1.590 (0.297)	**<.001**	1.517 (0.289)	**<.001**
mRS 4	3.645 (0.466)	**<.001**	4.734 (0.758)	**<.001**	3.850 (0.529)	**<.001**	1.631 (0.335)	**<.001**	1.511 (0.315)	**<.001**
mRS 5	2.462 (0.743)	**<.001**	4.564 (0.904)	**<.001**	2.809 (0.754)	**<.001**	1.828 (0.414)	**<.001**	1.572 (0.348)	**<.001**
Sex	-0.272 (0.138)	**.049**	-0.065 (0.163)	.689	-0.295 (0.137)	**.032**	-0.083 (0.132)	.527	-0.141 (0.126)	.261
Age	0.034 (0.006)	**<.001**	0.013 (0.007)	**.046**	-0.008 (0.006)	.153	-0.007 (0.005)	.183	-0.014 (0.005)	**.006**
Intercept	-5.273 (0.574)	**<.001**	-5.364 (0.863)	**<.001**	-2.596 (0.577)	**<.001**	-1.406 (0.451)	**.002**	-0.904 (0.428)	**.035**
Moderate problems										
mRS 1	1.676 (0.448)	**<.001**	1.335 (1.074)	.214	1.634 (0.622)	**.009**	1.604 (0.450)	**<.001**	0.829 (0.513)	.106
mRS 2	2.387 (0.432)	**<.001**	2.225 (1.023)	**.030**	3.298 (0.592)	**<.001**	2.606 (0.429)	**<.001**	1.988 (0.470)	**<.001**
mRS 3	3.320 (0.453)	**<.001**	4.119 (1.021)	**<.001**	4.604 (0.614)	**<.001**	3.021 (0.452)	**<.001**	2.677 (0.491)	**<.001**
mRS 4	4.434 (0.498)	**<.001**	6.024 (1.031)	**<.001**	5.154 (0.655)	**<.001**	3.537 (0.463)	**<.001**	3.176 (0.496)	**<.001**
mRS 5	3.480 (0.685)	**<.001**	5.724 (1.126)	**<.001**	4.573 (0.755)	**<.001**	3.935 (0.504)	**<.001**	2.974 (0.522)	**<.001**

Sex	0.024 (0.148)	.870	0.086 (0.208)	.680	-0.523 (0.152)	<.001	-0.243 (0.136)	.074	0.008 (0.153)	.960
Age	0.037 (0.006)	**<.001**	0.021 (0.008)	**.014**	-0.007 (0.006)	.240	-0.004 (0.006)	.426	0.000 (0.006)	.955
Intercept	-5.958 (0.633)	**<.001**	-6.689 (1.184)	**<.001**	-3.226 (0.728)	**<.001**	-2.757 (0.577)	**<.001**	-3.382 (0.641)	**<.001**
Severe problems										
mRS 1	29.974 (0.342)	**<.001**	-0.145 (0.000)	**<.001**	39.281 (0.000)	**<.001**	2.484 (1.034)	**.016**	-0.507 (1.418)	.720
mRS 2	30.932 (0.218)	**<.001**	14.990 (0.595)	**<.001**	54.134 (0.215)	**<.001**	3.318 (1.014)	**.001**	2.144 (1.027)	**.037**
mRS 3	32.421 (0.253)	**<.001**	18.322 (0.352)	**<.001**	56.355 (0.255)	**<.001**	4.328 (1.023)	**<.001**	3.736 (1.028)	**<.001**
mRS 4	34.238 (0.272)	**<.001**	21.121 (0.308)	**<.001**	58.055 (0.291)	**<.001**	5.023 (1.026)	**<.001**	4.455 (1.026)	**<.001**
mRS 5	34.459 (0.419)	**<.001**	21.982 (0.440)	**<.001**	57.906 (0.400)	**<.001**	5.651 (1.043)	**<.001**	4.264 (1.044)	**<.001**
Sex	-0.407 (0.216)	.060	0.180 (0.284)	.526	-0.247 (0.223)	.268	-0.289 (0.175)	.098	-0.408 (0.219)	.063
Age	0.037 (0.009)	**<.001**	0.019 (0.011)	.085	-0.020 (0.009)	**.021**	-0.002 (0.007)	.762	-0.008 (0.009)	.357
Intercept	-35.781 (0.561)	**<.001**	-22.014 (0.678)	**<.001**	-55.141 (0.519)	**<.001**	-4.679 (1.126)	**<.001**	-4.224 (1.174)	**<.001**
Extreme problems										
mRS 1	15.381 (0.000)	**<.001**	0.760 (0.000)	**<.001**	19.248 (0.000)	**<.001**	3.165 (0.000)	**<.001**	10.839 (0.862)	**<.001**
mRS 2	15.783 (0.000)	**<.001**	0.673 (0.000)	**<.001**	50.173 (0.341)	**<.001**	10.549 (0.355)	**<.001**	12.266 (0.386)	**<.001**
mRS 3	17.041 (0.000)	**<.001**	14.098 (0.812)	**<.001**	52.421 (0.353)	**<.001**	10.706 (0.615)	**<.001**	13.617 (0.440)	**<.001**
mRS 4	34.661 (0.360)	**<.001**	18.653 (0.427)	**<.001**	55.014 (0.318)	**<.001**	12.950 (0.374)	**<.001**	14.321 (0.409)	**<.001**
mRS 5	36.543 (0.431)	**<.001**	21.297 (0.489)	**<.001**	56.452 (0.400)	**<.001**	13.952 (0.445)	**<.001**	15.174 (0.416)	**<.001**

Sex	0.109 (0.298)	.715	0.617 (0.333)	.064	-0.313 (0.259)	.227	-0.383 (0.379)	.312	-0.459 (0.364)	.207
Age	0.027 (0.012)	**.023**	0.030 (0.014)	**.030**	-0.014 (0.010)	.173	-0.039 (0.014)	**.005**	-0.014 (0.014)	.329
Intercept	-36.018 (0.604)	**<.001**	-21.322 (0.821)	**<.001**	-52.795 (0.621)	**<.001**	-11.410 (0.762)	**<.001**	-15.132 (0.862)	**<.001**

Abbreviations: mRS=modified Rankin Scale; SE=standard error.
Note: mRS 0 is the reference category. Female sex is the reference category. Estimates with P<.05 are printed bold.

Table S7: Parameter estimates (SE) of the ordinal probit model

	Mobility		*Selfcare*		*Daily activities*		*Pain*		*Anxiety*	
**Parameter**	**Estimate (SE)**	**P-value**	**Estimate (SE)**	**P-value**	**Estimate (SE)**	**P-value**	**Estimate (SE)**	**P-value**	**Estimate (SE)**	**P-value**
mRS 1	0.945 (0.149)	**<.001**	0.638 (0.239)	**.007**	0.914 (0.168)	**<.001**	0.128 (0.128)	**<.001**	0.366 (0.132)	**.005**
mRS 2	1.301 (0.142)	**<.001**	1.118 (0.226)	**<.001**	1.694 (0.159)	**<.001**	0.119 (0.119)	**<.001**	0.807 (0.121)	**<.001**
mRS 3	1.787 (0.153)	**<.001**	2.041 (0.231)	**<.001**	2.376 (0.169)	**<.001**	0.133 (0.133)	**<.001**	1.292 (0.134)	**<.001**
mRS 4	2.689 (0.158)	**<.001**	3.160 (0.235)	**<.001**	3.200 (0.176)	**<.001**	0.137 (0.137)	**<.001**	1.591 (0.138)	**<.001**
mRS 5	3.919 (0.183)	**<.001**	4.342 (0.252)	**<.001**	4.142 (0.194)	**<.001**	0.149 (0.149)	**<.001**	1.653 (0.150)	**<.001**
Sex	-0.048 (0.057)	.400	0.062 (0.068)	.361	-0.157 (0.057)	**.006**	-0.122 (0.055)	**.025**	-0.098 (0.057)	.086
Age	0.015 (0.002)	**<.001**	0.009 (0.003)	**<.001**	-0.004 (0.002)	.059	-0.003 (0.002)	.123	-0.004 (0.002)	.119
Intercepts										
No problems | Slight problems	2.408 (0.221)	**<.001**	2.749 (0.299)	**<.001**	1.187 (0.225)	**<.001**	0.697 (0.194)	**<.001**	0.724 (0.200)	**<.001**
Slight problems | Moderate problems	3.067 (0.224)	**<.001**	3.447 (0.303)	**<.001**	1.954 (0.227)	**<.001**	1.323 (0.195)	**<.001**	1.408 (0.201)	**<.001**
Moderate problems | Severe problems	3.955 (0.229)	**<.001**	4.238 (0.309)	**<.001**	2.870 (0.230)	**<.001**	2.145 (0.198)	**<.001**	2.119 (0.205)	**<.001**
Severe problems | Extreme problems	4.768 (0.237)	**<.001**	4.984 (0.316)	**<.001**	3.564 (0.234)	**<.001**	3.223 (0.209)	**<.001**	2.864 (0.214)	**<.001**

Abbreviations: mRS=modified Rankin Scale; SE=standard error.

Note: mRS 0 is the reference category. Female sex is the reference category. The ordinal probit model was fitted using Bayesian functions; P-values must therefore be considered as approximate, as they are derived from a normal distribution (i.e. non-Bayesian approach). Estimates with P<.05 are printed bold.

Appendix V: Instructions pertaining R files

The multinomial logit model for each EQ-5D-5L dimension is uploaded as a supplemental R file. In this Appendix, we: 1) describe the used variables; 2) provide the reader with an R code sample, which may be used to apply the models to new data.

Variable description

The names of the response variables are (depending on the model/EQ-5D-5L dimension):

- eq5d5l_mobility
- eq5d5l_selfcare
- eq5d5l_dailyact
- eq5d5l_mood
- eq5d5l_pain

The predictor variables are:

- mrs_90d: an unordered factor variable describing the mRS score at 90 days.
- r_sex: a factor variable, with female sex as the reference category.
- r_age: a numeric variable.

R code sample

Provided that the reader has used the same names and variable types, the models can be used to predict EQ-5D-5L response level probabilities from a new dataset. The code below illustrates the usage of the models for this purpose. It also demonstrates how to calculate predicted utility values from the response level probabilities (please refer to Supplemental Appendix II for the used equation). The *tidyverse* package is needed to run the code.

```
prob_mlogit_dailyac <- predict(mlogit_activities, newdata = df_new, type = "probs")
prob_mlogit_mobility <- predict(mlogit_mobility, newdata = df_new, type = "probs")
prob_mlogit_selfcare <- predict(mlogit_selfcare, newdata = df_new, type = "probs")
prob_mlogit_mood <- predict(mlogit_mood, newdata = df_new, type = "probs")
prob_mlogit_pain <- predict(mlogit_pain, newdata = df_new, type = "probs")

df_new$pred_mlogit_dailyac_1 <- prob_mlogit_dailyac[,1]
df_new$pred_mlogit_dailyac_2 <- prob_mlogit_dailyac[,2]
df_new$pred_mlogit_dailyac_3 <- prob_mlogit_dailyac[,3]
df_new$pred_mlogit_dailyac_4 <- prob_mlogit_dailyac[,4]
df_new$pred_mlogit_dailyac_5 <- prob_mlogit_dailyac[,5]
```

```
df_new$pred_mlogit_mobility_1 <- prob_mlogit_mobility[,1]
df_new$pred_mlogit_mobility_2 <- prob_mlogit_mobility[,2]
df_new$pred_mlogit_mobility_3 <- prob_mlogit_mobility[,3]
df_new$pred_mlogit_mobility_4 <- prob_mlogit_mobility[,4]
df_new$pred_mlogit_mobility_5 <- prob_mlogit_mobility[,5]

df_new$pred_mlogit_selfcare_1 <- prob_mlogit_selfcare[,1]
df_new$pred_mlogit_selfcare_2 <- prob_mlogit_selfcare[,2]
df_new$pred_mlogit_selfcare_3 <- prob_mlogit_selfcare[,3]
df_new$pred_mlogit_selfcare_4 <- prob_mlogit_selfcare[,4]
df_new$pred_mlogit_selfcare_5 <- prob_mlogit_selfcare[,5]

df_new$pred_mlogit_mood_1 <- prob_mlogit_mood[,1]
df_new$pred_mlogit_mood_2 <- prob_mlogit_mood[,2]
df_new$pred_mlogit_mood_3 <- prob_mlogit_mood[,3]
df_new$pred_mlogit_mood_4 <- prob_mlogit_mood[,4]
df_new$pred_mlogit_mood_5 <- prob_mlogit_mood[,5]

df_new$pred_mlogit_pain_1 <- prob_mlogit_pain[,1]
```

```
df_new$pred_mlogit_pain_2 <- prob_mlogit_pain[,2]

df_new$pred_mlogit_pain_3 <- prob_mlogit_pain[,3]

df_new$pred_mlogit_pain_4 <- prob_mlogit_pain[,4]

df_new$pred_mlogit_pain_5 <- prob_mlogit_pain[,5]


df_new <- df_new %>%

  mutate(pred_mlogit = 1 -

         0_0354544 * pred_mlogit_mobility_2 - 0_0565962 * pred_mlogit_mobility_3 - 0_166003 * pred_mlogit_mobility_4 -
0_2032975 * pred_mlogit_mobility_5 -

         0_0381079 * pred_mlogit_selfcare_2 - 0_0605347 * pred_mlogit_selfcare_3 - 0_1677852 * pred_mlogit_selfcare_4 -
0_1677852 * pred_mlogit_selfcare_5 -

         0_0391539 * pred_mlogit_dailyac_2 - 0_0867559 * pred_mlogit_dailyac_3 - 0_1924631 * pred_mlogit_dailyac_4 -
0_1924631 * pred_mlogit_dailyac_5 -

         0_0658959 * pred_mlogit_pain_2 - 0_0919619 * pred_mlogit_pain_3 - 0_35993 * pred_mlogit_pain_4 - 0_4152142 *
pred_mlogit_pain_5 -

         0_069622 * pred_mlogit_mood_2 - 0_1445222 * pred_mlogit_mood_3 - 0_3563913 * pred_mlogit_mood_4 - 0_4206361 *
pred_mlogit_mood_5 -

         (1 - pred_mlogit_mobility_1 * pred_mlogit_selfcare_1 * pred_mlogit_dailyac_1 * pred_mlogit_pain_1 *
pred_mlogit_mood_1) * 0_0469233)
```
