# Supplementary material for: Mapping the mRS Into the EQ-5D-5L in Patients With Ischemic Stroke
Source: Stroke. 2026 Apr 29;57(6):1658–66. doi: 10.1161/STROKEAHA.126.055741 (PMC13196856; doi:10.1161/STROKEAHA.126.055741)
Supplement: Supplementary file 2 [file str-57-1658-s002.pdf]

Preferred Reporting Items for Studies Mapping onto Preference-Based Outcome Measures: The MAPS Statement

Section/topic	Item number	Recommendation	Reported on page number/line number
Title and abstract			
Title	1	Identify the report as a study mapping between outcome measures. State the source measure(s) and generic, preference-based target measure(s) used in the study	Page 1, line 1
Abstract	2	Provide a structured abstract including, as applicable: objectives; methods, including data sources and their key characteristics, outcome measures used and estimation and validation strategies; results, including indicators of model performance; conclusions; and implications of key findings	Page 3, line 32-56
Introduction			
Study rationale	3	Describe the rationale for the mapping study in the context of the broader evidence base	Page 5, line 65-79
Study objective	4	Specify the research question with reference to the source and target measures used and the disease or population context of the study	Page 5, line 80-83
Methods			
Estimation sample	5	Describe how the estimation sample was identified, why it was selected, the methods of recruitment and data collection, and its location(s) or setting(s)	Page 6, line 92-100
External validation sample	6	If an external validation sample was used, the rationale for selection, the methods of recruitment and data collection, and its location(s) or setting(s) should be described	Page 11, line 196-204
Source and target measures	7	Describe the source and target measures and the methods by which they were applied in the mapping study	Page 6, line 102-116
Exploratory data analysis	8	Describe the methods used to assess the degree of conceptual overlap between the source and target measures	Page 7, line 118-122
Missing data	9	State how much data were missing and how missing data were handled in the sample(s) used for the analyses	Page 6, line 99-100. Page 10, line 177-179
Modelling approaches	10	Describe and justify the statistical model(s) used to develop the mapping algorithm	Page 7, line 125-179

Section/topic	Item number	Recommendation	Reported on page number/line number
Estimation of predicted scores or utilities	11	Describe how predicted scores or utilities are estimated for each model specification	Page 10, line 181-194. Page 11, line 208-211
Validation methods	12	Describe and justify the methods used to validate the mapping algorithm	Page 10, line 181-211
Measures of model performance	13	State and justify the measure(s) of model performance that determine the choice of the preferred model(s) and describe how these measures were estimated and applied	Page 10, line 181-211
Results			
Final sample size(s)	14	State the size of the estimation sample and any validation sample(s) used in the analyses (including both number of individuals and number of observations)	Page 11, line 213-216
Descriptive information	15	Describe the characteristics of individuals in the sample(s) (or refer back to previous publications giving such information). Provide summary scores for source and target measures, and summarise results of analyses used to assess overlap between the source and target measures	Page 12, line 217-229. Table 1-4.
Model selection	16	State which model(s) is(are) preferred and justify why this(these) model(s) was(were) chosen	Page 12, line 231-243. Table 5 and 6.
Model coefficients	17	Provide all model coefficients and standard errors for the selected model(s). Provide clear guidance on how a user can calculate utility scores based on the outputs of the selected model(s)	Page 13, line 245-251. Table 7. Supplemental Appendix IV.
Uncertainty	18	Report information that enables users to estimate standard errors around mean utility predictions and individual-level variability	Page 13, line 245-251. Table 7. Supplemental Appendix IV.
Model performance and face validity	19	Present results of model performance, such as measures of prediction accuracy and fit statistics for the selected model(s) in a table or in the text. Provide an assessment of face validity of the selected model(s)	Page 12, line 231-243. Table 5 and 6.
Discussion			
Comparisons with previous studies	20	Report details of previously published studies developing mapping algorithms between the same source and target measures and describe differences between the algorithms, in terms	Page 14, line 260-300

Section/topic	Item number	Recommendation	Reported on page number/line number
		of model performance, predictions and coefficients, if applicable	
Study limitations	21	Outline the potential limitations of the mapping algorithm	Page 16, line 309-321
Scope of applications	22	Outline the clinical and research settings in which the mapping algorithm could be used	Page 16, line 306-308. Page 16, line 315-321.
Other			
Additional information	23	Describe the source(s) of funding and non-monetary support for the study, and the role of the funder(s) in its design, conduct and report. Report any conflicts of interest surrounding the roles of authors and funders	Page 17, line 332-338
